# *De novo* Biosynthesis of Biodiesel by *Escherichia coli* in Optimized Fed-Batch Cultivation

**DOI:** 10.1371/journal.pone.0020265

**Published:** 2011-05-23

**Authors:** Yangkai Duan, Zhi Zhu, Ke Cai, Xiaoming Tan, Xuefeng Lu

**Affiliations:** Key Laboratory of Biofuels, Qingdao Institute of Bioenergy and Bioprocess Technology, Chinese Academy of Sciences, Qingdao, China; University of Groningen, Netherlands

## Abstract

Biodiesel is a renewable alternative to petroleum diesel fuel that can contribute to carbon dioxide emission reduction and energy supply. Biodiesel is composed of fatty acid alkyl esters, including fatty acid methyl esters (FAMEs) and fatty acid ethyl esters (FAEEs), and is currently produced through the transesterification reaction of methanol (or ethanol) and triacylglycerols (TAGs). TAGs are mainly obtained from oilseed plants and microalgae. A sustainable supply of TAGs is a major bottleneck for current biodiesel production. Here we report the *de novo* biosynthesis of FAEEs from glucose, which can be derived from lignocellulosic biomass, in genetically engineered *Escherichia coli* by introduction of the ethanol-producing pathway from *Zymomonas mobilis*, genetic manipulation to increase the pool of fatty acyl-CoA, and heterologous expression of acyl-coenzyme A: diacylglycerol acyltransferase from *Acinetobacter baylyi*. An optimized fed-batch microbial fermentation of the modified *E. coli* strain yielded a titer of 922 mg L^−1^ FAEEs that consisted primarily of ethyl palmitate, -oleate, -myristate and -palmitoleate.

## Introduction

In order to meet the rapidly growing demand for transportation fuel and to achieve reduction of carbon dioxide emissions, development of renewable energy sources has become more and more urgent. Biodiesel, as one type of renewable energy, is an ideal substitute for petroleum-based diesel fuel and is usually made from plant oils or animal fats (triacylglycerides) by transesterification with methanol or ethanol resulting in fatty acid methyl esters (FAMEs) and fatty acid ethyl esters (FAEEs). However, the limited supply of bioresources to obtain triacylglycerides (TAGs) is becoming a major bottleneck for biodiesel production. The main reason is that vegetable oil feedstocks are also food sources and their planting is geographically limited. Microalgae are currently viewed as one of the most promising TAG feedstocks for biodiesel production. Although the productivity of these photosynthetic microorganisms greatly exceeds that of agricultural oleaginous crops, current microalgae production using the best available strains and cultivation methods has not yet become economically feasible for biodiesel production [1].

Lignocellulosic biomass is a relatively sustainable bioresource to make biofuels. In the past few decades, tremendous research and development efforts on cellulosic ethanol have resulted in significant advances and many technical problems have been solved [2], [3]. However, due to its low energy density, high vapor pressure and corrosiveness, bioethanol is not an ideal alternative to petroleum-derived fuels. Moreover, the high solubility of ethanol also results in toxicity to the microbes used to produce it [4]. Recently, technical routes have been developed to produce novel biofuel products with higher energy density and hydrophobic properties from lignocellulose-derived sugars. Liquid alkanes chemically identical to petroleum fuels can be made by both biosynthesis with genetically modified *E. coli* [5] and synthetic catalytic conversion [6]. Biosynthetic production of C4–C8 alcohols with straight or branched chains through manipulation of amino acid metabolic pathways in *E. coli* has been reported [7]. Thus, the production of novel biofuels from lignocellulosic biomass-derived sugars is a promising alternative to bioethanol.

Bioresource technologies for biodiesel production from lignocellulosic biomass, distinct from approaches using the oily fraction of biomass, can be developed through constructing non-native biosynthetic pathways of biodiesel molecules in microbial hosts. Direct microbial production of FAEEs in engineered *E. coli* was first reported by co-expressing genes coding enzymes for ethanol production from *Zymomonas mobilis* and the WS/DGAT gene encoding acyl-coenzyme A: diacylglycerol acyltransferase from *Acinetobacter baylyi* strain ADP1 [8]. However, biosynthesis of FAEEs in that work relied on the supplementation of exogenous fatty acids. In last couple of years, research focusing on overproduction of fatty acids by genetically engineering the fatty acid metabolic network towards providing precursors of fatty acid-based biofuels such as FAMEs, FAEEs, fatty alcohols, and fatty alkanes, has been extensively investigated in *E. coli*. The titer of 2.5 g L^−1^ day^−1^ free fatty acids was produced by an *E. coli* mutant strain in fed-batch fermentation with overexpression of acetyl-CoA carboxylase (ACC) and acyl-ACP thioesterase (TE) from *E. coli* as well as plant TE from *Cinnamomum camphorum*, and with deletion of *fadD*, which codes for fatty acyl-CoA synthase, the first step of fatty acid degradation [9]. An improved yield of fatty acids to a titer of 4.5 g L^−1^ day^−1^ has been demonstrated by the same group [10]. A very recent research publication in Nature has demonstrated *de novo* biosynthesis of FAEEs in *E. coli* by co-overproduction of ethanol and fatty acids in genetically engineered *E. coli*. In this work, several genetic engineering strategies were developed to improve fatty acid production of the ethanol-producing mutant strain to increase the yield of FAEEs, including overexpression of thioesterases, heterologous introduction of *fadD* gene from *Saccharomyces cerevisiae*, and the deletion of the *fadE* gene the encodes acyl-CoA dehydrogenase, leading to a yield of FAEEs of up to 674 mg L^−1^. Researchers have also demonstrated the feasibility of *in vivo* production of FAEEs from hemicellulose achieved *via* genetic engineering of the endoxylanase catalytic domain from *Clostridium stercorarium* and the xylanase from *Bacteroides ovatus* [11].

**Figure 1:**
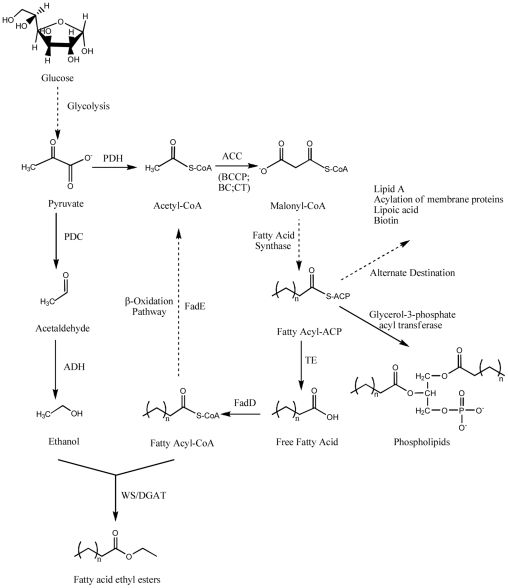
Constructed *de novo* biosynthetic pathway of fatty acid ethyl esters in *E. coli*. PDH: pyruvate dehydrogenase; ACC: acetyl-CoA carboxylase; BCCP: biotin carboxyl carrier protein; BC: biotin carboxylase; CT: carboxyltransferase; PDC: pyruvate decarboxylase; ADH: alcohol dehydrogenase; TE: thioesterase; FadD: fatty acyl-CoA synthase; FadE: acyl-CoA dehydrogenase; WS/DGAT: wax synthase/acyl-coenzyme A: diacylglycerol acyltransferase.

In our study, six distinct genetic alterations have been introduced into an *E. coli* BL21 (DE3) host strain (Fig. 1): (1) heterologous expression of the genes *pdc* and *adhB* from the ethanol-producing pathway in *Zymomonas mobilis*, coding pyruvate decarboxylase and alcohol dehydrogenase, respectively; (2) overexpression of *accBACD* genes coding acetyl-CoA carboxylase, which converts acetyl-CoA to malonyl-CoA, the first step of fatty acid biosynthesis that is proposed to be the rate-limiting step; (3) overexpression of the modified *tesA'* gene from *E. coli*, coding a leaderless version of thioesterase; (4) knockout of the *fadE* gene, coding acyl-CoA dehydrogenase that dehydrogenates fatty acyl-CoA, the second step of fatty acid degradation; (5) overexpression of the *fadD* gene from *E. coli*, coding a fatty acyl-CoA ligase that catalyzes the conversion of free fatty acids to fatty acyl-CoA; and (6) heterologous expression of the *atfA* gene from *A. baylyi*, coding the wax ester synthase/acyl-coenzyme A: diacylglycerol acyltransferase (WS/DGAT). Here, we also evaluated FAEE production in a scaled-up fed-batch fermentation, and optimized the nutritional and environmental conditions to improve the yield of FAEEs.

Compared to Steen *et al.*'s work on FAEE production reported in Nature [11], three significant differences were made in the current study. First, the *E. coli* mutant strain producing FAEE with over-expression of acetyl-CoA carboxylase that has been proved to be able to increase the rate of fatty acid biosynthesis in *E. coli* [12] was constructed in our study. Second, the effect of *fadE* deletion on FAEE production in both shake flask and scale-up fed-batch fermentation experiments was specially examined and analyzed in the report here. Third, we also evaluated FAEE production in a scale-up fed-batch fermentation, and optimized the nutritional conditions to improve the yield of FAEEs.

## Results and Discussion

### Shake flask experiment for *E. coli* strains

With the aim to achieve *de novo* biosynthesis of FAEEs in *E. coli*, plasmid pXT11, which contains *pdc* and *adhB* from *Zymomonas mobilis* ZM4, *fadD* from *E. coli*, and *atfA* from *Acinetobacter baylyi* ADP1, was constructed. When the *E. coli* strain harboring pXT11 was induced by 0.5 mM IPTG, no trace of FAEEs was detected by GC-MS. This is consistent with the previously reported results that FAEEs biosynthesis was dependent on the presence of a certain amount of free fatty acids and that the native level of fatty acyl-CoA was not sufficient to make FAEEs [8]. pMSD8 and pMSD15, containing *E. coli accBCDA* and *tesA'* expressing cassettes respectively, were proven to be effective in overproduction of free fatty acids in *E. coli* [9], [12]. In this study, pXT11, pMSD8, and pMSD15 were co-transformed into *E. coli* BL21 (DE3), and FAEEs were produced with the concentration of 34.6 mg L^−1^ by culturing the cells in shaking flasks with glucose as sole carbon source. To further increase the yield of FAEEs, the *fadE* gene, which encodes acyl-CoA dehydrogenase, was deleted to block degradation of fatty acyl-CoA, and it was observed that more than double the concentration of FAEEs, 77.5 mg L^−1^, was produced in the recombinant strain under the same culture conditions. This result indicates that overproduced fatty acyl-CoA, which is the substrate of acyl-coenzyme A: diacylglycerol acyltransferase that catalyzes the production of FAEEs, is essential for *de novo* biosynthesis of FAEEs in *E. coli*, and that the metabolic engineering strategy applied here is effective to achieve accumulation of fatty acyl-CoA.

### The effect of initial culture medium on FAEE production

Fig. S1a shows the production of different FAEEs using *E. coli* mutant strain BL21 (ΔfadE)/pXT11/pMSD8/pMSD15 fermented with three initial culture media. The maximum concentrations of FAEEs were 735, 922, and 328 mg L^−1^ (Table 1) when the cells were fermented in LB, 2LB, or 2LB+Phosphates medium, respectively. Thus, 2LB medium was selected as the initial culture medium for further experiments. It suggests that higher levels of nutrients in the initial culture medium have a positive effect for FAEE production and that excessive phosphates have negative effect.

**Table 1:** FAEE production of *E. coli* mutant strain BL21 (ΔfadE)/pXT11/pMSD8/pMSD15 under varied fed-batch fermentation conditions described in Materials and Methods.

*Categories of fermentation conditions*	*Varing conditions*	Maximum production of FAEE (mg L^−1^)
Initial culture medium	LB	735
	2LB	922
	2LB+phosphates	328
Feeding conditions	75 g/0.22 ml min^−1^	588
	100 g/0.22 ml min^−1^	922
	150 g/0.22 ml min^−1^	581
	200 g/0.11 ml min^−1^	464
Culture temperature	30°C	922
	25°C	652
Time for starting induction	4 hr	333
	11 hr	922
	16 hr	682

### The effect of feeding conditions on FAEEs production

The effects of glucose concentration (75, 100 and 150 g/750 ml) in the fed-batch fermentation of strain BL21 (ΔfadE)/pMSD8/pMSD15/pXT11 with the feed rate of 0.22 ml min^−1^ on the FAEEs production were evaluated. FAEEs were generated at 922 mg L^−1^ with 100 g glucose/750 ml feeding culture, while 581 and 588 mg L^−1^ FAEEs were produced in 75 g and 150 g glucose/750 ml feeding culture, respectively (Table 1, Fig. S1b). Moreover, the total FAEE concentration was only 464 mg L^−1^ when the glucose concentration was increased to 200 g glucose/750 ml feeding culture and the feed rate was decreased to 0.11 ml min^−1^, as shown in Table 1. This suggests that the concentration of glucose in the feeding culture and the feeding rate are highly related to FAEE production.

### The effect of culture temperature on FAEE production

The best FAEEs-producing strain in this work, *E. coli* BL21 (ΔfadE)/pMSD8/pMSD15/pXT11, contains three plasmids with three different origins. Temperature could affect the stability of these plasmids and also affect the production of FAEEs. When the strain was cultured at 37°C, no detectable FAEEs were found by GC-MS (data not shown). However, FAEEs were produced at 922 mg L^−1^ by the same strain when cultured at 30°C, and 652 mg L^−1^ of FAEEs were produced when the culturing temperature was decreased to 25°C (Table 1, Fig. S1c)).

### The effect of induction time point on FAEE production

In order to test the impact of induction time on FAEE production, three different time points in the early exponential stage were tested for induction of gene expression. As shown in Fig. S1d, when the culture was induced at an OD_600_ of 4, of 312 mg L^−1^ of FAEEs were produced at 18 h post-induction, and the total FAEE concentration did not change significantly during the next 20 h. When induced at an OD_600_ of 16, the concentration of FAEEs reached 682 mg L^−1^ at 32 h after the induction. The maximum FAEE yield of 922 mg L^−1^ was achieved 45 h after induction when the culture was induced at an OD_600_ of 11 (Table 1).

### Production of FAEEs in a fed-batch fermentation under the optimized conditions

To evaluate the performance of the fatty acid ethyl ester-overproducing strain in a large scale, a 5-L fed-batch fermentation was performed with the optimized cultivation conditions described above, in which fed-batch fermentations were carried out at 30°C with 2LB as the initial culture medium and 100 g glucose/750 ml as the feeding culture at a feed rate of 0.22 ml min^−1^, and the culture was induced at an OD_600_ of 11. Three recombinant *E. coli* strains, BL21 (DE3)/pXT11, BL21 (DE3)/pMSD8/pMSD15/pXT11, and BL21 (ΔfadE)/pMSD8/pMSD15/pXT11, were grown, and the concentrations of cells, ethanol, and FAEEs were measured.

**Figure 2:**
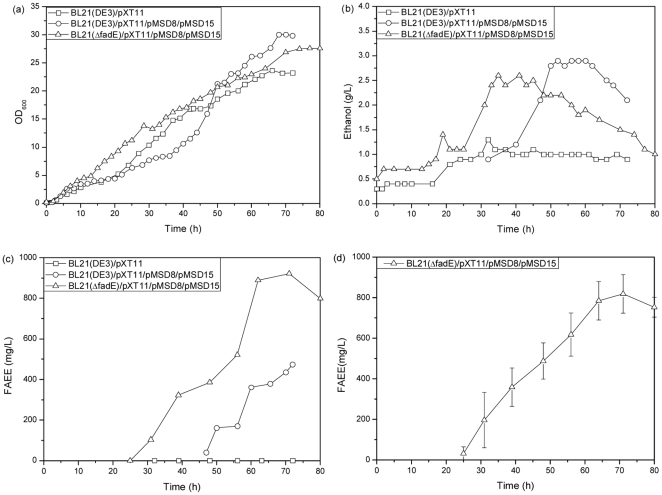
Analysis on fed-batch fermentations under the optimized conditions described in Materials and Methods. (a) Cell growth, (b) Ethanol production, (c) FAEE production of three *E. coli* mutant strains, BL21 (DE3)/pXT11 and BL21 (DE3)/pMSD8/pMSD15/pXT11 and BL21 (ΔfadE)/pMSD8/pMSD15/pXT11. (d) Three paralleled experiments for FAEE production of the strain BL21 (ΔfadE)/pMSD8/pMSD15/pXT11.

As shown in Fig. 2a, the growth of all three *E. coli* mutant strains was consistent with the logistic growth model. In the fed-batch fermentation, there was nearly no lag phase and cells grew directly into the exponential period, followed by a steady period after around 65–70 h. The maximum optical densities of the three strains, BL21 (DE3)/pXT11, BL21 (DE3)/pMSD8/pMSD15/pXT11, and BL21 (ΔfadE)/pMSD8/pMSD15/pXT11, reached around 24, 30 and 28 respectively. There is no significant difference among three cell growth profiles.

Ethanol production of the mutant strain BL21 (DE3)/pXT11, not producing FAEE, was clearly different than that in the two FAEE-producing strains (Fig. 2b). After induction, the concentration of ethanol increased slightly to around 1.3 g L^−1^ and then remained unchanged until the end of fermentation (Fig. 2b). It is reasonable that there is no ethanol consumption since there is no WS/DGAT enzyme in this strain to convert ethanol and no FAEE production in the strain. The ethanol production profile of the strain BL21 (DE3)/pMSD8/pMSD15/pXT11 was similar to that of the strain BL21 (ΔfadE)/pMSD8/pMSD15/pXT11 (Fig. 2b), and the maximum ethanol accumulations of both FAEE-producing strains were also similar (about 3 g L^−1^). The decrease of ethanol accumulations of the strain BL21 (ΔfadE)/pMSD8/pMSD15/pXT11 occurred earlier than that from the strain BL21 (DE3)/pMSD8/pMSD15/pXT11, and it is consistent with the FAEE production shown in Fig. 2c.

Based on the results obtained from the shake flask experiments, it was expected that no detectable FAEE would be obtained after induction during the fed-batch fermentation of BL21 (DE3)/pXT11 (Fig. 2c). FAEE production during fermentation of BL21 (DE3)/pMSD8/pMSD15/pXT11 and BL21 (ΔfadE)/pMSD8/pMSD15/pXT11 increased along with the decrease in ethanol production, and reached a maximum of 477 and 922 mg L^−1^ respectively, about 14- and 12-fold higher than the production in the corresponding shake flask experiments respectively. The fed-batch fermentation experiments also demonstrated the same doubling effect of the deletion of *fadE* on FAEE production as was observed in the shake flask experiments. To confirm FAEE production under the optimized fed-batch fermentation conditionsAnd thereby, two other parallelled fermentation experiments of fermentation were performed with the strain BL21 (ΔfadE)/pMSD8/pMSD15/pXT11 under the optimized culture conditions (Fig. 2d). The FAEE production, the glucose conversion efficiency and the specific productivity were calculated to be 818.50±94.64 mg L^−1^, 24.56±2.84 mg FAEE/g glucose and 0.46±0.11 mg L^−1^ OD^−1^ h^−1^ respectively.

### Composition of FAEEs during the optimized fed-batch fermentation

**Figure 3:**
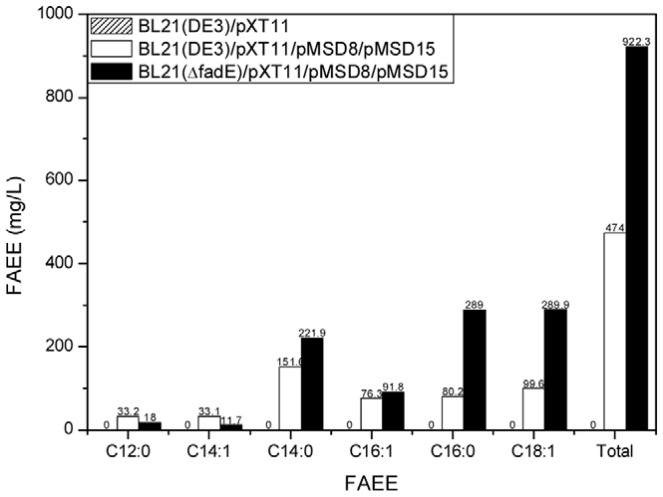
Composition of fatty acid ethyl esters with different carbon chain length and saturation degree during the optimized fed-batch fermentation of *E. coli* mutant strain BL21 (ΔfadE)/pMSD8/pMSD15/pXT11.

Fig. 3 illustrates the compositions of FAEEs produced in the fed-batch fermentation cultures of the three *E. coli* mutant strains. No detectable FAEE was found in the culture of BL21 (DE3)/pXT11. The fermentation of BL21 (DE3)/pMSD8/pMSD15/pXT11 produced 151.6 mg L^−1^ ethyl myristate (C14∶0; 32%) as the major constituent, with 99.6 mg L^−1^ ethyl oleate (C18∶1; 21%), 80.2 mg L^−1^ ethyl palmitate (C16∶0; 17%), 76.3 mg L^−1^ ethyl palmitoleate (C16∶1; 16%), 33.2 mg L^−1^ ethyl laurate (C12∶0; 7%), and 33.1 mg L^−1^ ethyl myristoleate (C14∶1; 7%) as the minor FAEEs observed. The fed-batch fermentation of BL21 (ΔfadE)/pMSD8/pMSD15/pXT11 strain under the same cultivation condition produced ethyl palmitate (16∶0; 31.3%) and ethyl oleate (18∶1; 31.4%) as the two major FAEE constituents, together with ethyl myristate (14∶0, 24.1%), ethyl palmitoleate (16∶1, 9.9%), and other two minor FAEE constituents, ethyl laurate (12∶0) and ethyl myristoleate (14∶1).

By comparison of the FAEE production of the two *E. coli* mutant strains, BL21 (DE3)/pMSD8/pMSD15/pXT11 and BL21 (ΔfadE)/pMSD8/pMSD15/pXT11, we found that blocking the degradation of fatty acyl-CoA through the deletion of the *fadE* gene encoding acyl-CoA dehydrogenase caused significant changes in the FAEE composition. Both mutant strains produced fatty acid ethyl esters with carbon chain length varying from 12 to 18. Neither of mutant strains produced ethyl stearate (18∶0), and ethyl laurate and ethyl myristoleate were minor constituents for both mutants. After *fadE* deletion, major products were ethyl palmitate (16∶0) and ethyl oleate (18∶1) rather than ethyl myristate (14∶0) in the mutant strain BL21 (DE3)/pMSD8/pMSD15/pXT11.

### Conclusions

In this study, a *de novo* biosynthetic pathway yielding fatty acid ethyl esters was constructed by genetically engineering *E. coli*. The fed-batch microbial fermentation was optimized with a maximum production of 922 mg L^−1^ FAEEs. Although the titer of FAEEs is low for further scaling-up, this work shows the feasibility and potential to utilize lignocellulosic biomass-derived sugars instead of oily biomass-derived TAGs to produce biodiesel. FAEE production could be significantly improved by increasing the fatty acid biosynthetic flux, balancing ethanol production and fatty acid synthesis, and engineering the WS/DGAT enzyme toward higher substrate specificity to ethanol and higher catalytic efficiency.

## Materials and Methods

### Enzymes, DNA Kits and Strains

Taq, Pfu DNA polymerase and T4 DNA ligase were purchased from Fermentas (Burlington, Canada) and all restriction enzymes were from Takara (Kyoto, Japan). Plasmid mini kits, PCR purification kits and gel extraction kits were ordered from Omega (Norcross, USA). *E. coli* strain BL21 (DE3) and DH5α were obtained from Takara (Kyoto, Japan).

### Plasmid construction

Detailed information about the plasmids used in this study is shown in Table 2. Plasmids pMSD8 and pMSD15 were kindly provided by Dr. John Cronan at University of Illinois at Urbana-Champaign, and pXL67 and pXL72 were generous gifts from Dr. Chaitan Khosla at Stanford University (USA). The *E. coli fadD* gene was excised from pXL72 *via* digestion with NdeI and SpeI, and cloned into the same sites of pXL67, resulting in pXT3. *Pdc* and *adh* genes from *Zymomonas mobilis* ZM4, coding pyruvate decarboxylase and alcohol dehydrogenase, were amplified from genomic DNA with primers pdc-up/pdc-down and adh-up/adh-down, and cloned into pXL67 resulting pXT4 and pXT5 respectively. The XbaI-SpeI double digested DNA fragment of pXT3, containing the *fadD* gene, was inserted into the SpeI site of pXL67, resulting in pXT9. The XbaI-SpeI double-digested DNA fragment of pXT5, containing the *adh* gene, was inserted into the SpeI site of pXT4, resulting in pXT10. Finally, the XbaI-SpeI double-digested DNA fragment of pXT10, containing both *pdc* and *adh* gene, was inserted into the SpeI site of pXT9, resulting in pXT11, in which four genes were assembled in the order of *atfA-fadD-pdc-adh* under control of the T7 promoter. Primers used here were listed in Table S1.

**Table 2:** Plasmids constructed and used in this study.

Plasmids	Relevant characteristic(s)	Source of reference(s)
pMSD8	Ap^r^; pFN476 derivative containing *E. coli accBCDA* genes; T7 promoter	[12]
pMSD15	Cm^r^; pACYA184 derivative containing *E. coli 'tesA* gene (without leading sequence); P_BAD_ promoter	[12]
pXL67	Ap^r^; pET28b derivative containing *Acinetobacter baylyi* ADP1 *atfA* gene; T7 promoter	Gift from Khosla
pXL72	Km^r^; pCR-Blunt vector derivative containing *E. coli fadD* gene	Gift from Khosla
pKD46	Ap^r^; Vector expressing the Red genes (γ, β, and *exo*) from phage λ under the arabinose-inducible *araB* promoter, *oriR101*, *repA101*(Ts)	[13]
pKD4	Ap^r^ Km^r^; Template plasmid for *kan* flanked by FRT sequences, *oriR6K*	[13]
pCP20	Ap^r^ Cm^r^; Helper plasmid, *FLP* λ *c*I857 λ P_R_ *repA*(Ts)	[14]
pXT9	Ap^r^; pET28b derivative containing *atfA-fadD* genes; T7 promoter	This study
pXT10	Ap^r^; pET28b derivative containing *pdc-adhB* genes; T7 promoter	This study
pXT11	Km^r^; pET28b derivative containing *atfA-fadD-pdc-adhB* genes; T7 promoter	This study

### *fadE* gene deletion from the chromosome of *E. coli* strain BL21 (DE3)

Homologous replacement was used to delete the *fadE* gene from the chromosome of *E. coli* strain BL21 (DE3) as described by Datsenko and Wanner (Datsenko and Wanner, 2000). Briefly, primers ΔfadE-up/ΔfadE-down were used to amplify the kanamycin resistance cassette (FRT-*kan*-FRT) from the template plasmid pKD46 (Table S1). The purified PCR product was subjected to DpnI digestion and electroporated into *E. coli* BL21 (DE3) carrying pKD46 (a helper plasmid that expresses the λ-Red functions). To test the insertion of the FRT-*kan*-FRT cassette, colony PCR was performed with primers k1/fadE-C1, k2/fadE-C2 and fadE-C1/fadE-C2, using kanamycin resistance transformants as templates. Subsequently, the kanamycin resistance gene was eliminated by using pCP20, a helper plasmid expressing the flippase (FLP) recombinase, and the kanamycin-sensitive transformants were also confirmed by colony PCR with primers k1/fadE-C1, k2/fadE-C2 and fadE-C1/fadE-C2 (Table S1).

### Cell transformation

*E. coli* BL21 (DE3) and BL21 (ΔfadE) competent cells were transformed by plasmids pMSD8, pMSD15, and pXT11, and cells were selected on solid LB plates containing carbenicillin (50 µg ml^−1^), kanamycin (50 µg ml^−1^), and chloramphenicol (17 µg ml^−1^).

### Shake flask cultures

Recombinant strains of *E. coli* were streaked onto LB agar plates with antibiotics (25 mg L^−1^ amplicillin, 25 mg L^−1^ kanamycin, and 17 mg L^−1^ chloramphenicol) and incubated at 37°C for 12–20 h. Single colonies were picked and inoculated into 10 ml of LB medium in 50 ml flasks, and the flasks were incubated at 37°C in a rotary shaker at 200 rpm for 12 h. The cells were collected by centrifugation at 5000 rpm for 1 min, resuspended into 50 ml of sterilized LB with 5 g L^−1^ glucose in 250 ml flasks, and shaken until an OD_600_ of 1.5–2 was reached. Arabinose was subsequently added into the culture to a final concentration of 0.4% for induction of the *araBAD* promoter. One hour later, IPTG was added to a final concentration of 0.5 M for induction of the T7 promoter. At about 20 h after induction, the culture was extracted for GC-MS analysis.

### Fed-batch fermentation

Fed-batch fermentation was performed in a 5-L fermentor (Biostat Bplus, Sartorius) with a working volume of 3 L. Three different LB formulations were evaluated as the initial medium, as shown in Table 1. 2LB medium contained 2% w/v tryptone, 1% w/v yeast extract and 1% w/v sodium chloride, while LB medium contained 1% w/v tryptone, 0.5% w/v yeast extract, 1% w/v sodium chloride, and the 2LB+Phosphates medium was the same as 2LB with the addition of 8.7 g/L K_2_HPO_4_ and 4.2 g/L Na_2_HPO_4_·12H_2_O. Appropriate antibiotics were added (see above). The stirrer speed was adjusted to 400 rpm. Unless stated otherwise, the pH was controlled at 7.5 by automatic addition of 2 M hydrochloric acid. The flow rate of air was maintained at 1 VVM (air volume per broth volume per minute). Inoculum was 5% (v/v) of overnight cultures (see above). Starting at an OD_600_ of 5–6, the culture was fed at a constant rate of 0.22 ml min^−1^ or 0.11 ml min^−1^ with sterilized glucose feed solutions containing 100 g yeast extract, 1.5 g magnesium sulfate, 0.75 g ammonium sulfate, and glucose at concentrations from 75 g to 200 g, all in a total volume of 750 ml (Table 1). Cells were induced at three different cultivation stages (OD_600_ values of 4, 11 or 16) by arabinose (final concentration of 0.4%) for pMSD15. After one hour IPTG (final concentration of 0.5 mM) was added to induce genes coded on pMSD8 and pXT11. Fermentation broth samples (∼20 ml) were collected at a series of time points and immediately kept at −80°C for fatty acid ethyl ester analysis.

### Analysis method

For analysis of fatty acid ethyl esters, 5 ml culture was mixed thoroughly with 5 ml of organic solvent containing chloroform and methanol (the ratio of organic solvents is 2∶1 by v/v), with 0.1 mg nonadecanoic acid methyl ester added as an internal standard. The organic phase was then collected and evaporated to dryness under a nitrogen atmosphere, and redissolved in 2 ml of n-hexane. Samples were analyzed by GC-MS (Thermo Scientific ITQ 1100™ GC/MSn system, USA) using a single quadrupole MS with an electron impact ionization source. The TR-5 MS GC column was 30 m in length, with 0.25 mm ID and 25 mm film thickness. The following temperature program was applied: 1 min at 40°C, 15 min ramp to 280°C, and constant at 280°C for 10 min. The quantification of fatty acid methyl esters was achieved by reference to the internal standard.

Ethanol concentration was determined using a biosensor (SBA-40C) from Biology Institute of Shandong Academy of Science (Jinan, China).

## Supporting Information

Figure S1 FAEE production of *E. coli* mutant strain BL21 (ΔfadE)/pXT11/pMSD8/pMSD15 under varied fed-batch fermentation conditions: (a) varying initial culture medium; (b) varying feed glucose and feeding rate; (c) varying cultivation temperature; (d) varying induction time point.
(TIF)

Table S1 Strains and primers used in this study.
(DOC)
