# Supplementary figures and images for: De novo Biosynthesis of Biodiesel by Escherichia coli in Optimized Fed-Batch Cultivation
Source: PLoS One. 2011 May 23;6(5):e20265. doi: 10.1371/journal.pone.0020265 (PMC3100327; doi:10.1371/journal.pone.0020265)

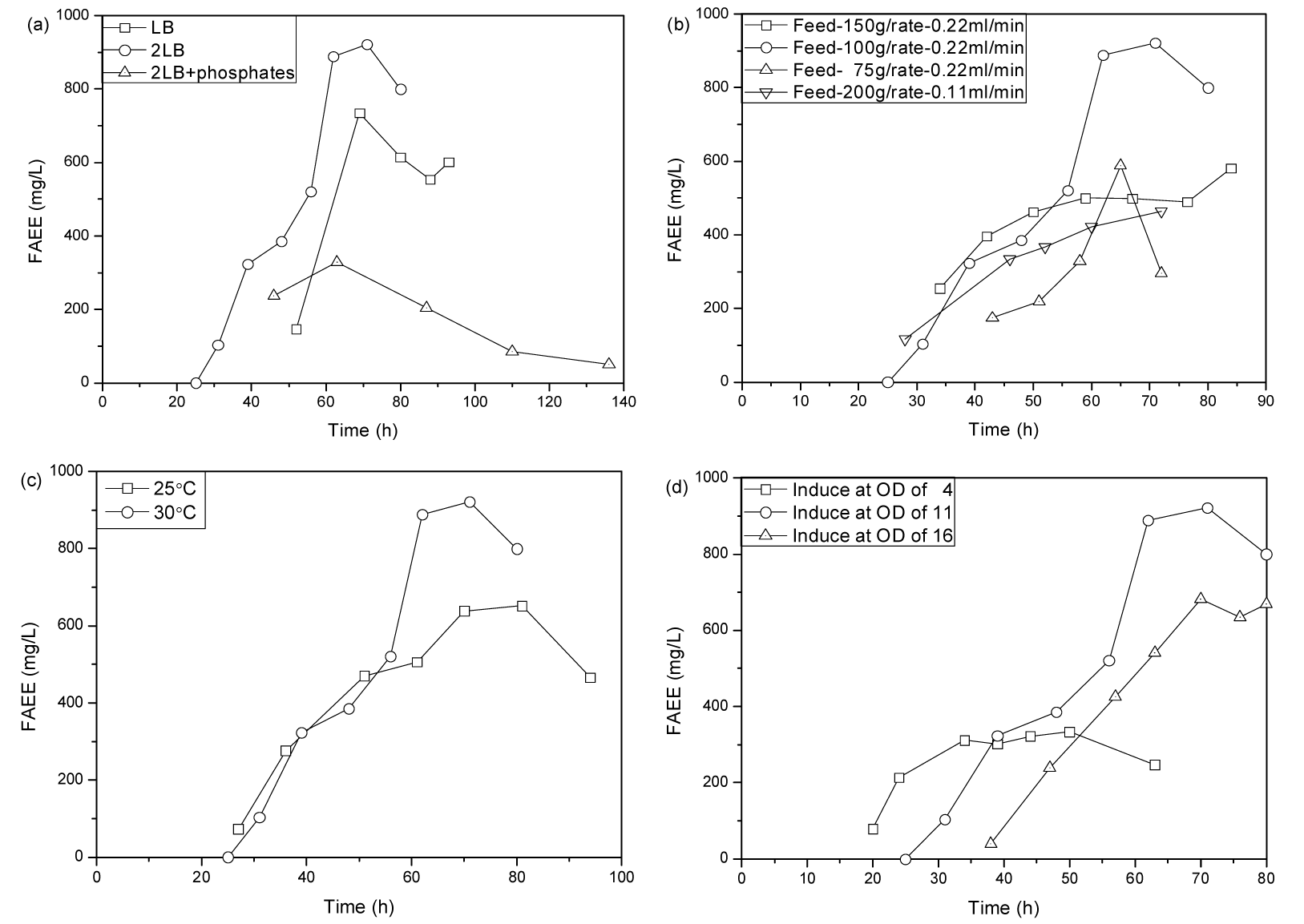

Supplement: Figure S1 — FAEE production of E. coli mutant strain BL21 (ΔfadE)/pXT11/pMSD8/pMSD15 under varied fed-batch fermentation conditions: (a) varying initial culture medium; (b) varying feed glucose and feeding rate; (c) varying cultivation temperature; (d) varying induction time point. (TIF) [file pone.0020265.s001.tif]
