# Supplementary material for: De novo Biosynthesis of Biodiesel by Escherichia coli in Optimized Fed-Batch Cultivation
Source: PLoS One. 2011 May 23;6(5):e20265. doi: 10.1371/journal.pone.0020265 (PMC3100327; doi:10.1371/journal.pone.0020265)
Supplement: Table S1 — Strains and primers used in this study. (DOC) [file pone.0020265.s002.doc]

**Table S1** Strains and primers used in this study.

Strains used in this study

| Strains | Genotype and/or relevant property | Source of reference(s) |
| --- | --- | --- |
| DH5α | F^–^ *endA*1 *thi-*1 *recA*1 *relA*1 *gyrA*96 *deoR* *phoA supE*44 Φ80d/*lacZ*ΔM15 Δ(*lacZYA-argF*)U169 *hsdR*17(*r*_K_^-^ *m*_K_^+^), λ^–^ | Takara |
| BL21(DE3) | F^–^ *ompT hsdS_B_*(*r*_B_^-^ *m*_B_^-^) *gal* (λ *c I* 857 *ind*1 *Sam*7 *nin*5 lacUV5-T7 gene1) *dcm*(DE3) | Takara |
| BL21(DE3-ΔfadE) | BL21(DE3), but *ΔfadE* | This study |

Primers used in this study

| Primers | Sequence | Source of reference(s) |
| --- | --- | --- |
| adh-up | GGCATATGGCTTCTTCAACTTTTTATATTCCTTTCGTCAACGAAATGGGCG | This study |
| adh-down | GCGCTCGAG TTAGAAAGCGCTCAGGA | This study |
| pdc-up | GGCATATGAGTTATACTGTCGGTACCTATTTAGCGG | This study |
| pdc-down | AACCTCGAGCTAGAGGAGCTTGTTAACAGGC | This study |
| ΔfadE-up | ATGATGATTTTGAGTATTCTCGCTACGGTTGTCCTGCTCGGCGCGTTGTTC GTGTAGGCTGGAGCTGCTTC | This study |
| ΔfadE-down | TTACGCGGCTTCAACTTTCCGCACTTTCTCCGGCAACTTTACCGGCTTCGTCATATGAATATCCTCCTTAG | This study |
| fadE-C1 | TAAAGCTGTCTGCTAACAGGACC | This study |
| fadE-C2 | GTGATGAAAGGCGGTACTTATAC | This study |
| k1 | CAGTCATAGCCGAATAGCCT | [13] |
| k2 | CGGTGCCCTGAATGAACTGC | [13] |
